# HEALTHY PEOPLE 2030 AND HEALTHY AGING

**DOI:** 10.1093/geroni/igac059.718

**Published:** 2022-12-20

**Authors:** Yen Lin

**Affiliations:** Office of Disease Prevention and Health Promotion, Rockville, Maryland, United States

## Abstract

The US Department of Health and Human Services, Office of Disease Prevention and Health Promotion (ODPHP) sets science-based health promotion and disease prevention objectives for the nation with 10-year targets through the Healthy People Initiative. In addition to objectives specific to older adults, many of our population-based objectives in other condition-specific topics also contain data for older adults. Healthy People also developed the Department of Health and Human Service's Social Determinants of Health model. This presentation will highlight ODPHP's policy initiatives and campaigns specifically for older adults, including the Dietary Guidelines for Americans 2020-2025, Move Your Way © for Older Adults and provide an update on the Physical Activity Guidelines Midcourse Report on Older Adults.

